# Conidial surface proteins at the interface of fungal infections

**DOI:** 10.1371/journal.ppat.1007939

**Published:** 2019-09-12

**Authors:** Matthew G. Blango, Olaf Kniemeyer, Axel A. Brakhage

**Affiliations:** 1 Department of Molecular and Applied Microbiology, Leibniz Institute for Natural Product Research and Infection Biology (HKI), Jena, Germany; 2 Department of Microbiology and Molecular Biology, Institute for Microbiology, Friedrich Schiller University Jena, Jena, Germany; McGill University, CANADA

## Introduction to spores—They are all around us

Spores are small, usually unicellular reproductive units produced to propagate genetic material by prokaryotic and eukaryotic microbes, including algae and protozoa, lower vascular plants, and even a subset of animals [1]. In prokaryotes, the endospores generated by some members of the phylum Firmicutes evolved for stress resistance and long-term survival of extreme environments [2]. In the lower vascular plants like ferns and some mosses, unicellular spores located underneath the leaves of nonflowering plants, similar to multicellular seeds in fruits or flowers, transfer genetic material to the next generation and often into new environments [3]. In eukaryotic microbes, the unicellular slime molds and fungi produce spores during their life cycle or in response to environmental stress [4, 5]. Although often overlooked, fungi play an essential role in the environment as saprotrophs, mycorrhizal symbionts, and even, in some cases, as parasites or pathogens. Fungi are typically nonmotile, but production and dissemination of spores by both marine and terrestrial fungi offer a mechanism for wider genetic dispersal [6]. Water currents, plants, and animals all disperse fungal spores, but the most commonly considered form of spore transport is wind, where astonishingly high fungal spore fluxes have been observed in terrestrial ecosystems (513 spores per m^2^ s^1^) [6, 7]. Locally higher fluxes are even present during meteorological events, like thunderstorms or high wind events, and in particular ecosystems, like cropland, which show measured fluxes of approximately 2,500 spores per m^2^ s^1^ [6, 7]. The presence of spores in the air column is commonly linked to respiratory diseases, as in the case of thunderstorm asthma [8, 9]. Fungal spores are not only a problem in humans but are also a major source of disease in insects, plants, and other animals. Our shifting climate is expected to lead to increasing exposure to spores and subsequent fungal infections due to the ubiquity of fungi in the environment [10].

**Fig 1:**
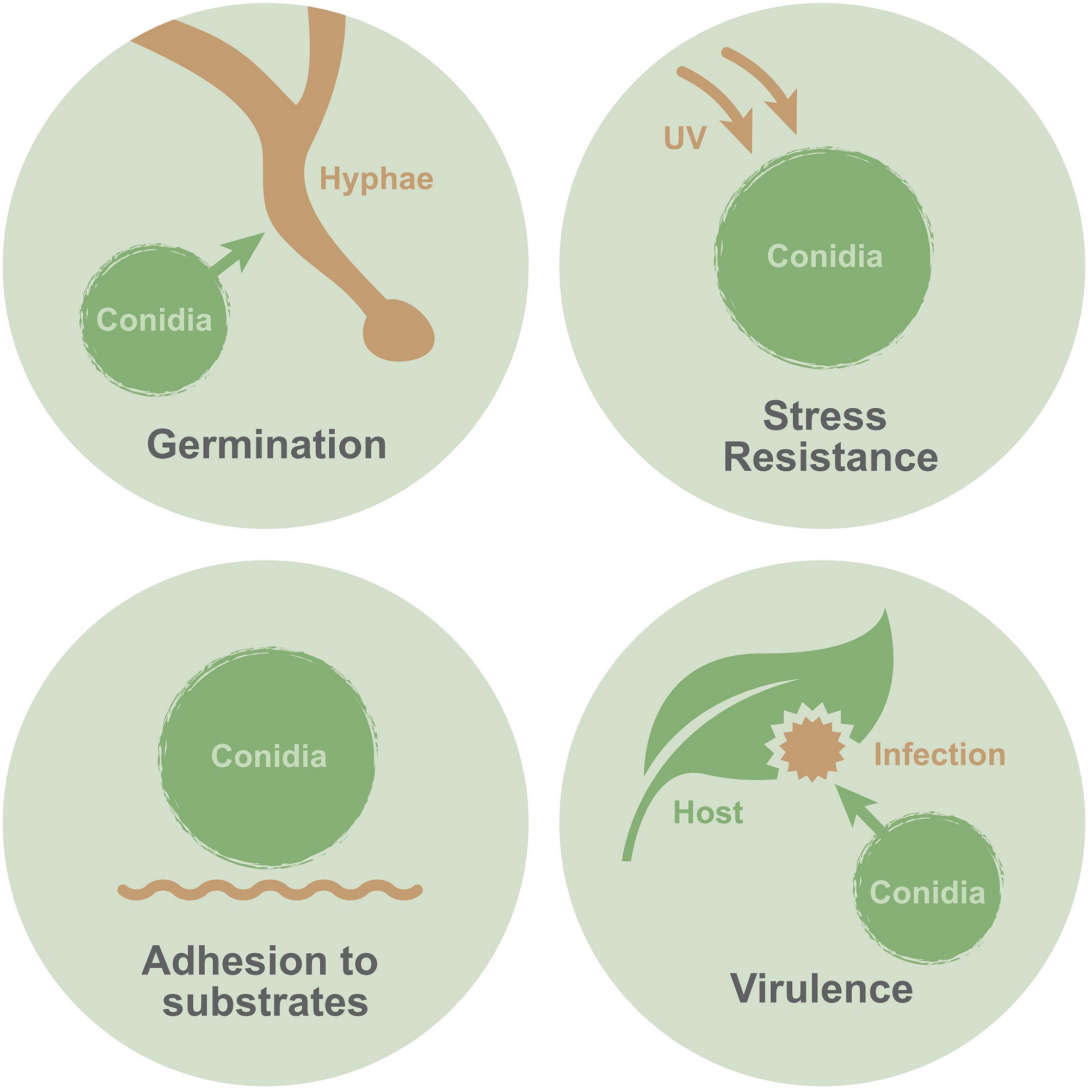
Conidial surface proteins have many roles. Proteins on the surface of conidia are involved in a variety of important functions. In particular, these proteins contribute to germination, stress resistance, adhesion to substrates, and virulence.

Fungi produce a huge diversity of spores as part of their life cycle for propagation, in response to stress, and for niche establishment. These spores help define the fungi, and spores are typically named after the reproductive structure that produces them, with spores formed by sexual reproduction named “ascospores” in the Ascomycota or “basidiospores” in the Basidiomycota, for example. Spores formed by asexual reproduction are also defined according to their mode of production: arthrospores and chlamydospores differentiate directly from an entire mycelium or hyphal compartment, respectively; sporangiospores form inside sporangia; motile flagellated zoospores are released from zoosporangia; and conidia are exogenously produced on stalklike conidiophores [5]. For brevity, we focus here only on conidia, which are nonmotile, walled, haploid cells generated by mitosis from the parent fungal cell and a major source of infection [1, 5]. We discuss the role of fungal conidia in infection of mammalian, plant, and insect hosts and expound on the major functions of conidial surface proteins in facilitating hydrophobicity, adhesion, and virulence in this diverse set of organisms (Fig 1).

## Conidial hydrophobins aid in dispersal and contribute to immune evasion

The best-studied examples of conidial surface proteins are the widely conserved hydrophobins of filamentous fungi. Conidial hydrophobins are cysteine-containing functional amyloid proteins that drive hydrophobicity and promote air buoyancy [11, 12]. Biophysical characterizations have revealed two classes of hydrophobins; Class I hydrophobins form a characteristic rodlet structure often present on conidia, whereas Class II hydrophobins assemble amphiphilic films at air–water interfaces [13]. The hydrophobins are found in a variety of fungal genera, including both saprophytes and pathogens of the Ascomycetes (Class I and II hydrophobins) and Basidiomycetes (Class I hydrophobins), such as *Aspergillus*, *Cladosporium*, *Penicillium*, *Neurospora*, *Magnaporthe*, *Schizophyllum*, *Phanerochaete*, and *Beauveria* [11, 12, 14, 15]. The cellular localization of hydrophobins is quite variable. In some cases, these proteins are found only on the conidia, while in other organisms, they are present on mycelia or even secreted [16, 17]. In the important human pathogen *Aspergillus fumigatus*, the hydrophobins are tightly regulated, with the hydrophobic barrier dismantled during germination to aid in nutrient exchange and growth [18]. The *A*. *fumigatus* hydrophobins, along with closely related orthologs, have been shown to contribute to immune evasion of conidia by masking host Dectin-1– and Dectin-2–dependent immune recognition of fungal spores [19, 20]. The *A*. *fumigatus* hydrophobins, in particular, the RodA protein, also inhibit platelet activation during infection, providing an advantage for the fungus in establishing infection in an immunocompromised host [21]. In line with these findings, the frequency of human antigen–specific T cells that recognize conidial proteins is lower than for those that target mycelial antigens, again reiterating the capacity of *A*. *fumigatus* conidia to evade the immune response [22]. Interestingly, this saprophytic mold is thought to have developed these immune evasion strategies in the environment and not in the host [23], potentially in response to predation by soil-dwelling amoebae [24–26]. Conversely, the human host had to evolve to efficiently remove these ubiquitous conidia while limiting hyperreactivity that would damage host tissues [19].

Other fungal pathogens have more obviously evolved with their hosts, as in the case of the entomopathogenic fungus *Beauveria bassiana*, which causes white muscardine disease in a wide range of arthropods [27]. In *B*. *bassiana*, the hydrophobins not only increase water-mediated dispersal but also facilitate adhesion to the insect cuticle for invasion [28]. Intriguingly, the *B*. *bassiana* hydrophobins promoted virulence in insect injection experiments and were even hypothesized to lack a role in immune evasion [28]. If confirmed, these findings would be in stark contrast to the immune evasion phenotype in *A*. *fumigatus*, reaffirming that fungi have evolved multiple functions for hydrophobins during infection. *B*. *bassiana* is a particularly interesting case due to its large host range. It will be interesting to learn in the future if the hydrophobins of *B*. *bassiana* are perhaps used differently depending on the particular arthropod host infected.

Along with providing hydrophobicity, facilitating adhesion, and aiding evasion of host immune responses, the conidial hydrophobins also influence interactions with other microorganisms. For example, in the entomopathogenic and nematophagous fungus *Clonostachys rosea*, hydrophobin deletion strains exhibited increased tolerance to fungal secondary metabolites produced by growth competitors, consistent with a down-regulation of these proteins during interaction [29]. This result suggests that the surface proteome of conidia must be tightly regulated to respond appropriately to changing environmental conditions; however, more work is required to fully understand the role of hydrophobins in interactions between microbes.

## Conidial surface proteins mediate adherence to hosts

The hydrophobins are not alone on the conidial surface, and in fact, many other proteins contribute to substrate adhesion. The best examples come from human pathogens, for which multiple studies have described *A*. *fumigatus* proteins contributing to adhesion. Interestingly, in *A*. *fumigatus* hyphae, the exopolysaccharide galactosaminogalactan is the major mediator of hyphal adhesion; however, this molecule is absent from conidia, suggesting that other factors likely contribute to adhesion [30, 31]. Early studies linked adherence to the hydrophobin, RodA, and the allergen, AspF2 (reviewed in [32]), but it was quickly realized that other proteins must also contribute to adhesion. The glycophosphatidylinositol-anchored protein CspA was next shown to aid in surface adhesion [33], likely through indirect effects on cell wall architecture [32, 34]. The FleA lectin is another example of a conidial surface protein that mediates adhesion to the host, particularly to airway mucins [35]. Detection of FleA by the host is essential for proper clearance of conidia by macrophages and resolution of the infection [35]. In more recent work using comparative phenotypic and transcriptomic analyses, additional adhesion molecules were predicted, including a haemolysin-like protein that potentially has a moonlighting function on the conidial surface [36]. These predictions remain preliminary, and further experimentation will be required to prove that these proteins are both on the surface and contributing to adhesion, but collectively, these studies assert that a large number of proteins contribute to *A*. *fumigatus* adhesion in the host. In the mucoralean fungus *Rhizopus oryzae*, the CotH proteins found on the surface of spores promote adhesion and invasion by acting as ligands for glucose-regulated protein 78 (GRP78) on the surface of endothelial cells, similar to the examples from *A*. *fumigatus* [37].

In other organisms, the contribution of conidial proteins to adhesion is less clear. Early work with the nematode pathogen *Drechmeria coniospora* indicated that a conidial adhesive substance sensitive to protease treatment is produced at a so-called conidial “adhesive bud” [38]. This adhesive facilitates adherence to the host chemosensory organs of a range of nematode species; however, the exact proteins important for adhesion remain unidentified [38]. In the entomopathogen, *Hirsutella satumaensis*, another example of an adhesive, known in this case as mucilage, is produced that coats the spore and aids in attachment to insect cuticles. When this layer is removed, the spores show decreased adherence but not complete abolishment of attachment, suggesting that other factors might also contribute to substrate adhesion [17]. We can learn a bit more about these adhesive substances from *Venturia inaequalis*, the cause of apple scab disease, which produces conidia that adhere to leaves, again using a secreted spore tip glue [39]. In this case, the interaction relies on hydrophobic interactions with the spore surface in the presence of water to facilitate attachment [39]. One final example of a spore adhesive secretion comes from the rice blast fungus, *Magnaporthe oryzae*, in which apically secreted spore tip mucilage mediates adhesion during infection of plant leaves [40]. In addition to this mucilage, at least one conidial hydrophobin, MPG1, also seems to contribute to surface adhesion under certain experimental conditions [41, 42]. MPG1 also helps retain localized activity of cutinase 2, an enzyme important for entry into host plant tissue [12]. Collectively, these studies highlight the complicated nature of conidial adhesion as a process influenced by both classical adhesion molecules and complex adhesive secretions; much work remains to fully understand the diversity of adherence mechanisms employed by fungal conidia and the specificity of these interactions with host receptors.

## Surface proteins contribute to virulence

We have already learned that surface proteins often contribute to adherence, stress resistance, and immune evasion, so it is perhaps not surprising that there are also many cases in which conidial proteins directly influence the outcome of infection. The *R*. *oryzae* CotH protein, important for adherence, has also been linked to virulence. Heterologous production of the CotH protein in nonpathogenic *Saccharomyces cerevisiae* facilitated invasion of host cells via the GRP78 receptor, indicating that CotH is genetically sufficient to confer invasion to a nonpathogenic organism [37]. In addition, an *R*. *oryzae cotH* deletion strain exhibited decreased invasion, reduced epithelial cell damage, and partially attenuated virulence in a mouse model of mucormycosis [37]. Intriguingly, antibodies targeting CotH were shown to be protective against infection in the mouse model, suggesting potential as an immunotherapeutic agent in the future [37].

In *A*. *fumigatus*, genetic deletion of the RodA hydrophobin or the CcpA surface protein resulted in increased host immune activation, indicating a function in masking conidial antigenicity [19, 43]. Unlike RodA, CcpA is essential for virulence in a corticosteroid-induced immunosuppression mouse infection model; however, the exact mode of action and full importance in human patients remain unknown [43]. Another important protein on the surface of *A*. *fumigatus* conidia is the Mep1p metalloprotease, which is released from spores in the mammalian lung to cleave host complement proteins and enhance infection, similar to the Alp1p serine protease released from mycelia for the same purpose [44]. The thaumatin-like protein CalA contributes to *A*. *fumigatus* invasion of epithelial cells through interaction with α_5_β_1_ integrin on the host surface [45]. It is important to note that CalA, like many conidial surface proteins, is produced not only on swollen conidia but also on hyphae [45].

The entomopathogenic fungus and cause of green muscardine disease, *Metarhizium anisopliae*, commonly infects a wide range of arthropods and relies on numerous conidial surface protein activities to promote infection. In particular, a surface protein fraction was shown to contain protease, chitinase, lipase, as well as peroxidase, superoxide dismutase, and phospholipase C activities [14]. The conidia of *M*. *anisopliae* appear to drive infection by providing spores with tools to manage stressful situations and establish a new niche in the host. In the future, more work will be required to determine the contribution of each of these activities to infection; however, the observed phospholipase C activity is consistent with known virulence factor activities from other systems [14]. Collectively, these reports reveal the importance of conidial surface proteins in promoting infection from a variety of important fungal pathogens but again highlight the need for additional studies to establish both the rules and the peculiarities of fungal pathogenesis.

## Surface proteins have potential biomedical and industrial applications

The ultimate goal of defining the conidial surface proteome is to improve our understanding of fungal pathogenesis and identify novel targets for early detection or immunotherapy. In particular, detection of fungal conidia from environmental samples might provide an early warning to those suffering from lung conditions like asthma or chronic obstructive pulmonary disease, in which patients show a heightened susceptibility to allergic exacerbations due to fungal sensitization [46]. The hydrophobins are one putative class of proteins with potential diagnostic value, along with specific proteins like the *A*. *fumigatus* CcpA protein or the *R*. *oryzae* CotH proteins, for example [37, 43]. A key to diagnosis will be finding biomarkers that are surface-localized under a diverse array of conditions, a feature that might prove difficult. Recent work in *A*. *fumigatus* suggests that the surface proteome of conidia is quite dynamic and environment dependent, making diagnosis through a single surface biomarker, like the hydrophobins, extremely challenging [43]. Proteins like *A*. *fumigatus* CalA are of great interest, as they are on the surface of multiple morphotypes of the fungi, including swollen conidia and hyphae [45]. We also have to take into account the ubiquity of fungal conidia, which makes contamination of highly sensitive diagnostics from the local environment an ever-present issue.

As surface proteins offer a likely first point of contact between pathogen and host, their biology offers interesting insight into fungal pathogenesis. In the long term, we hope that these surface proteins can be leveraged in some way to aid in the resolution of these devastating infections. Although conidial-specific proteins are unlikely to function as strong vaccine candidates, a potential alternative application would be as immunostimulatory molecules to provoke fungal-specific serological responses or T-cell activation. Studies of the conidial surface proteome will also likely aid in the selection of candidates for passive or adoptive transfer experiments of immunoglobulins or T cells, respectively, by helping us to understand the surface localization of these proteins across germination. We also suspect that additional proteins remain undiscovered that trigger allergy or aid in evading immune detection. We urge the fungal community to learn from other systems, like the prokaryotes, in which additional activities have already been ascribed to surface proteins, as with the kinase activity of the CotH proteins that phosphorylate extracellular proteins to aid in germination of the endospore [47]. In addition to utilizing the knowledge gained from studies of surface proteins in therapeutics, we also have to be open to alternate uses for these discoveries. For example, numerous reports suggest that the hydrophobins can be used to coat surfaces for both biomedical and industrial applications due to their unique properties as functional amyloids [13, 48]. In conclusion, conidial surface proteins play an integral role at the interface between normal fungal function and pathogenesis while offering a wealth of potential biomarkers and novel therapeutic targets.
